# MiR-142-3p is a RANKL-dependent inducer of cell death in osteoclasts

**DOI:** 10.1038/srep24980

**Published:** 2016-04-26

**Authors:** Jezrom B. Fordham, Katherine Guilfoyle, Afsar Raza Naqvi, Salvador Nares

**Affiliations:** 1 Department of Periodontics, University of Illinois at Chicago, Chicago, Illinois, USA; 2 University of North Carolina at Chapel Hill, School of Dentistry, Department of Periodontics, 385 S. Columbia St., Chapel Hill, NC, 27599, USA

## Abstract

MicroRNA are small, non-coding, single-stranded RNAs that are estimated to regulate ~60% of the human genome. MiRNA profiling of monocyte-to-osteoclast differentiation identified miR-142-3p as a miRNA that is significantly, differentially expressed throughout osteoclastogenesis. Enforced expression of miR-142-3p via transient transfection with miR-142-3p mimic inhibited cell-to-cell contact and fusion, decreased protein kinase C alpha expression, and ultimately reduced cell viability. miR-142-3p was also identified as significantly differentially expressed during monocyte-to-macrophage differentiation and overexpression of miR-142-3p prevented their conversion to osteoclasts. Furthermore, the inhibitory effect of miR-142-3p on osteoclastogenesis extended to the conversion of a third osteoclast precursor cell type- dendritic cells. These results demonstrate miR-142-3p to be a negative regulator of osteoclastogenesis from the 3 main precursor cell types: monocytes, macrophages and dendritic cells. Importantly, decreased survival was dependent upon both miR-142-3p expression and RANK-signaling, with no harmful effects detected in the absence of this combination. As such, miR-142-3p represents a novel target for the selective removal of osteoclasts by targeting of osteoclastogenic pathways.

Dysregulated osteoclast (OC) function is a pathological component in a wide range of diseases affecting bone, such as osteoporosis, osteopetrosis, and metastatic breast cancer invasion of bone. As such, defining the regulatory processes coupled to OC development and function are of great therapeutic interest. Significant progress has been made in elucidating the cellular and molecular components governing bone metabolism; however, the impact of epigenetic regulation on OC development and function is not fully understood.

MicroRNAs (miRNA) have emerged as key players in the post-transcriptional regulatory mechanism of gene expression. MiRNA represent small, non-coding, single-stranded RNAs that regulate a plethora of cellular processes^1^, and previous studies involving the silencing of DGCR8 and Dicer (essential for miRNA synthesis) have confirmed the importance of miRNA for osteoclastogenesis^2^. The importance of miRNA in OC differentiation and function is also evidenced by decreased activation of the OC transcription factors c-fos, PU.1, MITF and NFATc1 when DGCR8, Dicer or Ago2 (essential factors for miRNA homeostasis) are silenced^2^. To date, few miRNAs have been identified that regulate OC differentiation. However, an increasing body of evidence clearly indicate that miRNAs are operative at the commitment stage of osteoclastogenesis^3^.

Protein kinase C alpha (PKCα) is one of several serine/threonine kinases that regulate important cellular functions including cell differentiation, apoptosis, and LPS signaling^4^. PKC-dependent pathways have been reported to play a key role in RANKL-induced OC differentiation, expression of OC-specific genes, and OC function^5^. Pharmacological inhibition of PKCα significantly reduces several hallmarks of osteoclastogenesis, including decreased cell size, a lower average number of nuclei per OC, and the average ratio of cell area per nucleus^6^. Functionally, PKCα inhibition reduces the ability of OC to resorb bone^4^.

To date only a few miRNAs have been reported to target PKCα including miR-200b^7^, miR-203^8^, pre-miR-24-2^9^, and miR-15a^10^. However, these reports highlight miRNA targeting of PKCα during carcinogenesis/metastasis but not during normal bone metabolism. Previously, we have demonstrated miR-142-3p mediated inhibition of MΦ and DC phagocytosis via direct regulation of PKCα^11^. Here we identify downregulation of miR-142-3p as a requirement for monocyte-to-OC differentiation, as well as macrophage and DC conversion to OC.

## Results

### MiRNA profiling during osteoclastogenesis reveals differential expression of novel miRNAs

**Figure 1:**
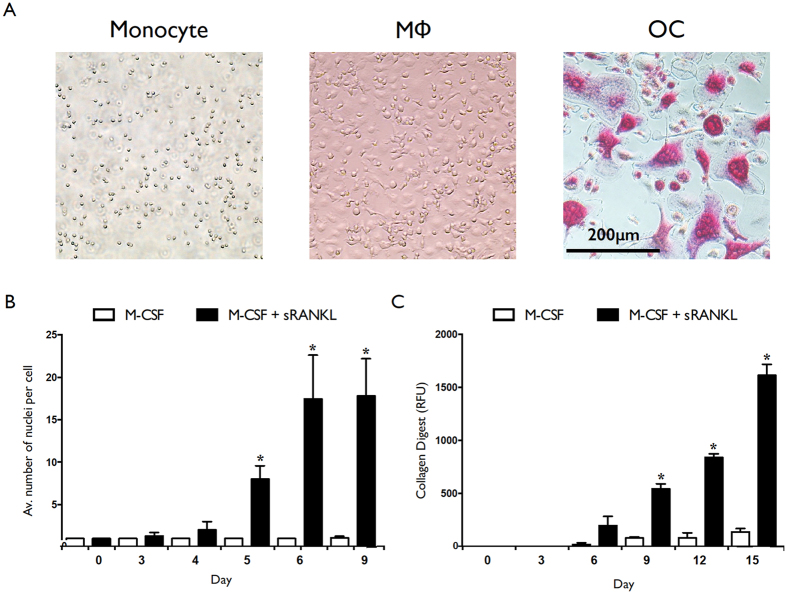
Phenotypic confirmation of monocyte-to-osteoclast and monocyte-to-macrophage differentiation. Human CD14+ monocytes were cultured in the presence of M-CSF and sRANKL, or M-CSF only. (**A**) Representative images of monocytes (day 0), macrophages (day 7) and OC (day 9; TRAP stained). (**B**) Average number of nuclei per cell for M-CSF + sRANKL (filled bars) and M-CSF only (empty bars) cultures over time. (**C**) Collagen degradation of M-CSF + sRANKL (filled bar) and M-CSF only (open bar) cultures over time. Data are presented as mean and SD, or representative images, from 4 biological donors. *p < 0.05 relative to monocyte (day 0) controls. P values were calculated using the Student’s t-test.

**Figure 2:**
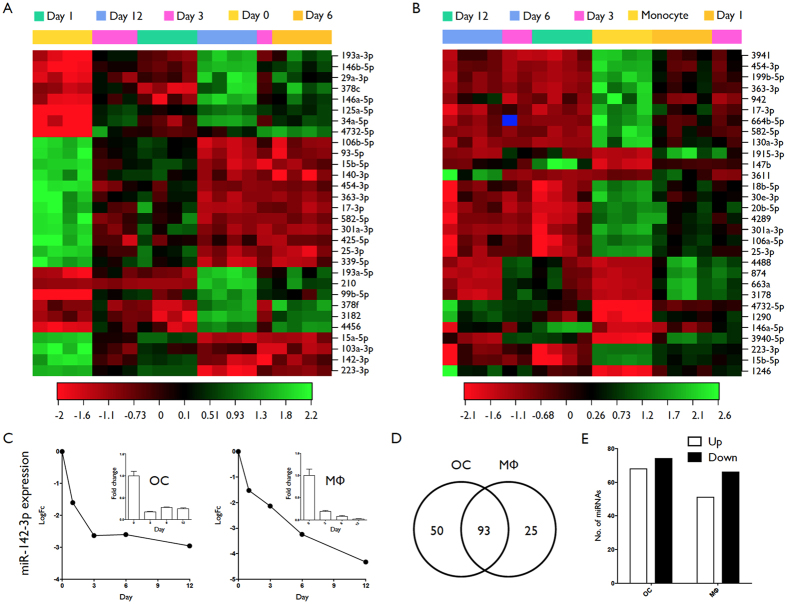
miRNA profiling of monocyte to osteoclast differentiation. Human CD14+ monocytes were cultured in the presence of M-CSF+ sRANKL, or M-CSF only, and total RNA was isolated at day 0, 1, 3, 6 & 12. (**A**) Heat map diagram of hierarchical clustering of samples and relative miRNA expression (top 30) for OC differentiation. (**B**) Heat map diagram of hierarchical clustering of samples and relative miRNA expression (top 30) for macrophage differentiation. (**C**) Expression of miR-142-3p over time for OC (left) and macrophage (right) as identified by microarray (main) and RT-PCR (insert). (**D**) Venn diagram of significantly differentially expressed miRNAs identified during monocyte-to-osteoclast and monocyte-to-macrophage differentiation. (**E**) Bar chart comparing the total number of upregulated (open bar) and downregulated (filled bar) miRNAs in both groups. Data are presented as mean and SD from 4 biological donors. P values were calculated by ANOVA and adjusted using the Benjamini Hochberg method.

Human CD14+ monocytes were isolated from healthy human donors and differentiated into OC using a combination of recombinant human (rh) M-CSF and sRANKL, or macrophages by the addition of M-CSF alone. Representative images of monocytes (day 0), macrophages (day 7) and osteoclasts (TRAP stained, day 9) are presented in Fig. 1A. Osteoclastogenesis was confirmed by quantification of average number of nuclei per cell (Fig. 1B) and ability to digest collagen (Fig. 1C). Total RNA was collected at days 1, 3, 6, and 12 of differentiation and miRNA profiling was performed by microarray. An expression analysis of variance over time was performed and heat maps showing the relative expression level of the top 30 differentially expressed miRNAs are shown in Fig. 2 (OC differentiation is shown in Fig. 2A; macrophage differentiation in Fig. 2B). Analysis was performed using the complete-linkage method together with the euclidean distance measure. miRNAs identified as differentially expressed during osteoclastogenesis included novel miRNAs such as miR-142-3p, as well miRNAs known to be involved in osteoclastogenesis- for example miR-223. Expression of miR-142-3p over time for OC (left) and macrophage (right) is presented in Fig. 2C. Inserts are validations of microarray results performed by RT-PCR. A total of 143 and 117 miRNAs were identified as significantly (ANOVA; p = <0.05) differentially expressed during OC and macrophage differentiation, respectively (Fig. 2D). A total of 50 miRNAs were unique to OC differentiation, 25 miRNAs were unique to macrophage differentiation, and 93 miRNAs were present in both groups (Fig. 2D). Analysis of the direction of expression change revealed that miRNA downregulation was slightly favored in both processes (Fig. 2E).

### Overexpression of miR-142-3p inhibits monocyte to OC differentiation

**Figure 3:**
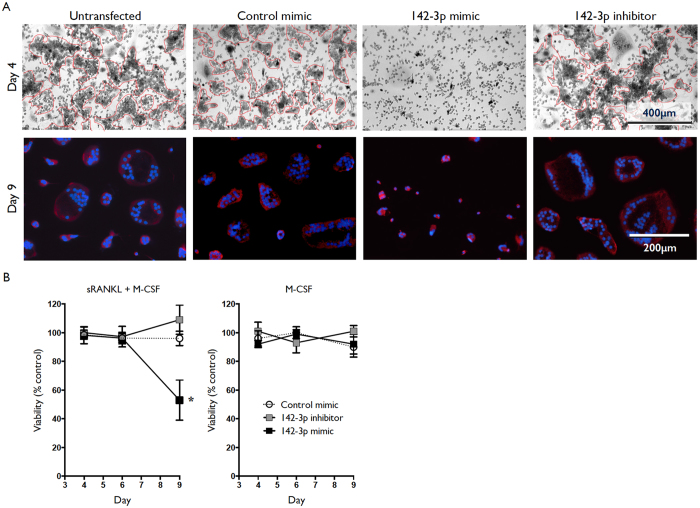
Increased cell death of osteoclast precursors by overexpression of miR-142-3p. Human CD14+ monocytes were cultured in the presence of M-CSF and sRANKL, or M-CSF only. Cultures were left untreated or transfected with miR-142-3p mimic, miR-142-3p inhibitor, or control mimic at day 2. (**A**) Brightfield images of differentiating osteoclasts (M-CSF and sRANKL) were taken at day 4 (top) and images of cells stained with Hoescht (nuclei; blue) and MitoTracker (mitochondria, red) were taken at day 9 (bottom). Red lines highlight areas of significant cell-cell contact. (**B**) Viability of M-CSF plus sRANKL, and M-CSF only, cultures assessed at different time-points by MTS assay, relative to untransfected cells. Filled black squares represent miR-142-3p mimic transfected cells, filled grey squares represent miR-142-3p inhibitor transfected cells, and empty circles represent control mimic transfected cells. Data are presented as mean and SD, or representative images, from 5 biological donors. *p < 0.05 relative to untreated control cells. P values were calculated using the Student’s t-test.

The functional significance of miR-142-3p expression during osteoclastogenesis was assessed using transient transfection methods to introduce miR-142-3p mimic, inhibitor, or control mimic (miRNA mimic possessing no interaction with any human 3′ UTR) to differentiating cultures. Cells were transfected at day 2 with miR-142-3p mimic, inhibitor, or control mimic. As previously reported, our transfection efficiency for these cells was approximately 90%^11^,^12^. OC cultures overexpressing miR-142-3p displayed greatly reduced clustering and fusion events relative to untreated, control mimic, or miR-142-3p inhibitor, at day 4 of culture. (Fig. 3A, top). Red lines highlight areas of significant cell-cell contact that is an immediate precursor to fusion. By day 9, OC cultures overexpressing miR-142-3p contained far fewer cells and displayed a reduced number of multi-nucleated cells (Fig. 3A, bottom). There was a trend for greater clustering, fusion and higher average number of nuclei per cell in miR-142 inhibitor transfected cells, but this was not statistically significant (data not shown). Cell viability was assessed by MTS assay and this demonstrated a miR-142-3p mimic-dependent increase in cell death; however, this effect was only present from day 6 onwards, indicating that the reduced ability to cluster and fuse observed at day 4 was not due to reduced cell number (Fig. 3B, left). Furthermore, this decrease in viability was not present in the corresponding control cells in which sRANKL was omitted, demonstrating that increased cell death was sRANKL dependent (Fig. 3B, right). This is consistent with our previously reported effects of miR-142-3p overexpression/knock-down during monocyte-to-macrophage differentiation, in which we described no significant alteration of cell viability in the presence of M-CSF, GM-CSF, or GM-CSF plus IL-4 signaling^12^.

### miR-142-3p mediated down-regulation of PKCα expression contributes to reduced OC survival.

**Figure 4:**
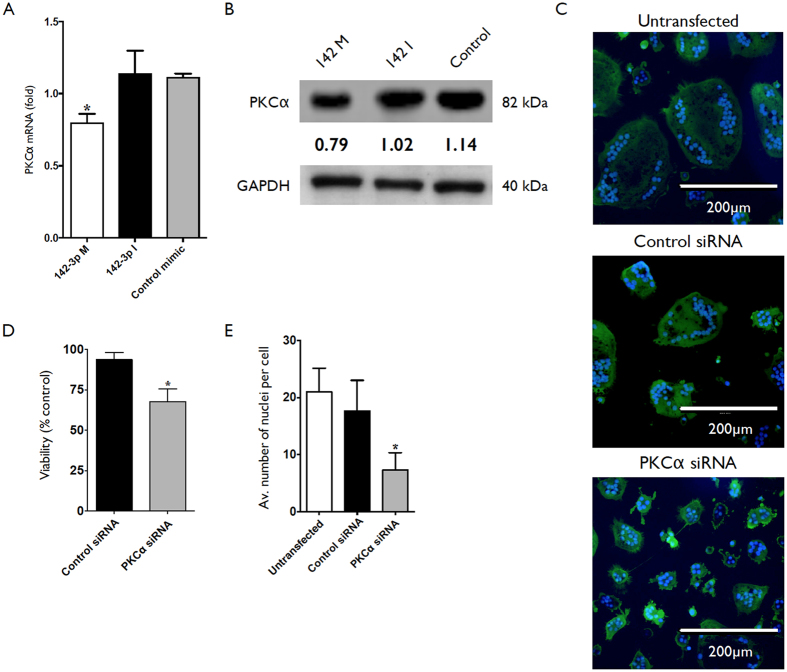
Phenotype and PKCα expression in osteoclast cultures transfected with miR-142-3p mimic or PKCα siRNA. Human CD14+ monocytes were cultured in the presence of M-CSF and sRANKL. (**A**) Cultures were left untreated or transfected with miR-142-3p mimic, miR-142-3p inhibitor, or control mimic. PKCα expression was assessed by qRT-PCR at day 4, or (**B**) by western blot at day 6. (**C**) Cultures were left untreated or transfected with PKCα siRNA, or control siRNA, at day 2. Images of cells stained with Hoescht (nuclei; blue) and LysoTracker (lysosomes, green) taken at day 9. (**D**) Viability of cultures at day 9 assessed by MTS assay. Expression is relative to untransfected controls. (**E**) Average number of nuclei per cell of cultures transfected with PKCα siRNA, control siRNA, or untransfected. Data are presented as mean and SD, or representative images, from 3 biological donors. *p < 0.05 relative to untreated control cells. P values were calculated using the Student’s t-test.

Day 2 differentiating OC were transfected with miR-142-3p mimic, inhibitor, or control mimic and PKCα protein and mRNA levels were assessed on day 6 and day 4, respectively. Results were normalized to GAPDH expression for both assays. Cultures transfected with miR-142-3p mimic displayed reduced PKCα at the level of mRNA and protein (Fig. 4A,B, respectively). Day 2 differentiating OC were also transfected with siRNA for PKCα or control siRNA. OC cultures transfected with PKCα siRNA displayed reduced size (Fig. 3C), reduced viability (Fig. 3D) and reduced average nuclei per cell (Fig. 3E).

### miR-142-3p expression prevents the generation of OC from macrophage and DC

**Figure 5:**
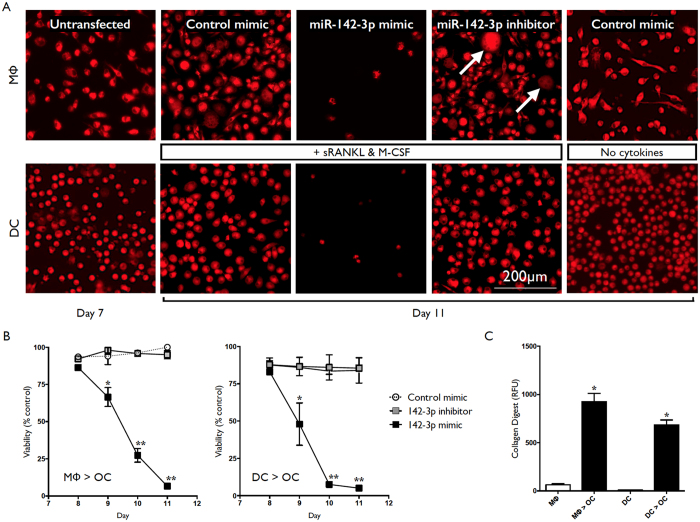
Inhibition of macrophage and DC conversion to OC with miR-142-3p overexpression. Human CD14+ monocytes were cultured in the presence of M-CSF or GM-CSF plus IL-4 for differentiation into macrophages and DC, respectively. Macrophage and DC were transfected at day 7 with miR-142-3p mimic, miR-142-3p inhibitor, or control mimic, and culture conditions were substituted for OC-differentiating conditions. (**A**) Representative images of macrophage and DC at day 7, at day 11 with transfection and OC-differentiating conditions, and day 11 negative controls (control mimic transfected, absence of OC-differentiating conditions). Cells were stained using MitoTracker (mitochondria, red). (**B**) Viability of macrophage-to-OC and DC-to-OC cultures from day 8 to day 11 assessed by MTS assay. (**C**) Collagen degradation of macrophage-to-OC and DC-to-OC cultures (untransfected) assayed at day 14. Data are presented as mean and SD, or representative images, from 3 biological donors. *p < 0.05 relative to untreated control cells. P values were calculated using the Student’s t-test.

Human CD14+ monocytes were differentiated into macrophages using M-CSF, or into DC using GM-CSF and IL-4, as described previously^11^,^12^. These cells displayed all the phenotypic properties of macrophages and (immature) DC by day 7 of culture^11^,^12^. Day 7 cultures were left untransfected or transfected with miR-142-3p mimic, miR-142-3p inhibitor, or control mimic. 18 hrs later culture media was substituted for OC-differentiating media (RPMI, M-CSF+ sRANKL). At day 11, cultures combining transfection with control mimic or miR-142-3p inhibitor and OC-differentiating conditions displayed increased size, and in the case of MΦ to OC conversion, the presence of giant cells (highlighted by white arrows) (Fig. 5A). In contrast, cultures combining transfection with miR-142-3p mimic and OC-differentiating conditions displayed very few cells (Fig. 5A). Assessment of cell viability from day 8 to day 11 by MTS assay revealed a miR-142-3p mimic-dependent, sRANKL-dependent decrease in viability from day 9 through to day 11 (Fig. 5B). Validation of the protocol for MΦ and DC conversion to OC was assessed by collagen digest. At 14 days, untransfected macrophage and DC cultures cultured in OC-differentiating conditions from day 8 displayed OC-like morphology and possessed the ability to degrade collagen (Fig. 5C; MΦ > OC & DC > OC bars). Cultures maintained in the absence of OC-differentiating conditions did not significantly degrade collagen (Fig. 5C; MΦ & DC bars).

## Discussion

Osteoclasts are giant, multi-nucleated cells central to bone remodeling, being uniquely equipped for resorption and acting in concert with bone-forming osteoblasts to regulate bone mass. We profiled miRNA expression during monocyte-to-OC differentiation and found 143 miRNAs that were differentially expressed over time. These included miRNAs, such as miR-223, whose role in osteoclastogenesis has been well-characterized^13^, as well as novel miRNAs only identified due to advances in microarray technology, such as miR-4732-5p. Over a third of these miRNAs were specific to monocyte-to-osteoclast differentiation, being absent from monocyte-to-macrophage differentiation. The majority of miRNAs identified by profiling of monocyte-to-OC & monocyte-to-macrophage differentiation were similarly regulated in both groups. This is unsurprising given the nature of these processes, which include a common precursor cell, the presence of M-CSF signaling, and the same commitment to increased cell size and viability.

The identification of previously reported miRNAs, such as miR-223, provides further support both for their role in osteoclastogenesis, and for the validity of the novel miRNAs identified, such as miR-142-3p. miR-223 has been reported to regulate osteoclastogenesis by modulation of PU.1, NFI-A and M-CSFR circuitry, including c-Fos and CREB^14^, and while miR-142-3p is not predicted to target any of these, we show that miR-142-3p similarly regulates osteoclastogenesis. This occurs via a mechanism that includes the targeting of PKCα, reduced cell-to-cell contact and fusion, and reduced cell viability. This phenomenon is RANKL-dependent and extends to macrophage and DC conversion to OC.

PKC is a well-established promoter of osteoclastogenesis. PKC stimulates RANKL expression in osteoblasts/stromal cells and PKC inhibitors reduce OC differentiation and function^15^. miR-142-3p expression is significantly downregulated over the first 3 days of monocyte-to-OC differentiation with diminished expression maintained from day 3 through to day 12. This pattern was also present in the control group of monocyte-to-macrophage differentiation. We have previously reported miR-142-3p expression to be similarly decreased during monocyte-to-DC differentiation. Here we show miR-142-3p to be a negative regulator of both *de novo* and ‘second-hand’ osteoclastogenesis, and postulate that such an effect is likely to be more efficacious (with regards to therapy) than a single-pathway approach to targeted modulation. Furthermore, while the fact that increased miR-142-3p expression correlates with increased cell death may be considered a weakness, we believe this must be tempered by the observation that it is RANKL-dependent. Monocyte-to-macrophage differentiation performed using the same conditions, but in the absence of sRANKL, demonstrated no such decrease in cell viability. Also, miR-142-3p mimic transfected macrophages or DCs were not adversely affected when exposed to M-CSF in the absence of sRANKL. Thus, in this context, miR-142-3p may be considered a RANKL-dependent inducer of cell death.

While we have demonstrated the direct effect on PKCα by miR-142-3p, it is likely that other interactions may contribute to our findings. A number of studies have indicated an important role for the PKC pathway in osteoclastogenesis. PKC exists in multiple isoforms including α, β, δ, ε, ζ, θ, ι and λ variants. These isoforms display tissue-specific expression profiles and they are believed to possess common and independent mechanisms of function. Multiple PKC isoforms, including α, β, δ, ε and ζ, have been shown to be expressed during OC formation^16^. The pattern of expression of these isoforms has been reported to ‘flip’ during the related processes of monocyte-to-macrophage and monocyte-to-DC and this has been linked to the decreased propensity for cell death that accompanies monocyte commitment to differentiation. Previously, we reported an inverse correlation of miR-142-3p with PKCα mRNA levels and confirmed PKCα as a novel target of miR-142-3p^11^. PKCα signaling has also been shown to regulate apoptosis via altered expression of apoptosis-related genes, such as BCL2 and BCL2L1. These two molecules are also predicted targets of miR-142-3p and future work will provide insight into the contribution of miR-142-3p targeting of PKCα relative to its targeting of components downstream of PKCα signaling. Future work will also include screening the miRNAs identified here for their possible regulation of PKC isoforms.

Osteoclastogenesis requires cytoskeletal rearrangement including reorganization of the microtubule network and polarization of the actin network^17^. Osteoclastogenesis is also regulated by PKCα expression and this molecule is known to mediate signaling pathways that result in cytoskeletal rearrangement^18^,^19^. Overexpression of miR-142-3p inhibited cell-to-cell contact, clustering and fusion events, a property that we believe is attributed to reduced PKCα-mediated rearrangements of the microtubule and actin networks; however, this does not provide a rationale for the induction of cell death that we observed. We postulate that the observed decrease in PKCα results in decreased expression of anti-apoptotic factors downstream of PKCα, which are known to include BCL2 and BCR-ABL^20^,^21^. Our bioinformatic analysis has also revealed several potential interactions of miR-142-3p with BCL2 family members, including BCL2 and BCL2L1, as well as signaling molecules upstream of the PKCα pathway, such as phospho-inositide 3 kinase (PI-3K). The relative direct and indirect contributions of miR-142-3p target regulation on the observed phenotype will be interrogated. Given the promiscuity of miRNA-3′ UTR interactions, and the predilection of miRNAs for regulating biology at the network level rather than single target modulation, we would anticipate the identification of multiple interactions that together explain the phenotypic effect described in this article.

## Materials and Methods

### Primary human monocyte isolation and OC generation

Freshly prepared buffy coats were collected from healthy donors (n ≥ 3) (Sylvan N. Goldman Oklahoma Blood Institute), CD14+ monocytes were obtained by density gradient centrifugation and magnetic bead isolation as described previously^11^,^12^. Briefly, PMBCs were purified using Ficoll Paque^TM^ (GE Healthcare) based density centrifugation. PBMCs were incubated with magnetically labeled CD14 beads (Miltenyi Biotech) according to manufacturer’s instructions. Monocyte purity and viability was >95%, as determined by flow cytometry. For OC differentiation, monocytes were plated at 4 × 10^6^/ml in RPMI supplemented with penicillin (100U/ml) and streptomycin (100 μg/ml). After 2 hours the media was substituted with media containing 10% FBS (Life Technologies), recombinant human M-CSF (50 ng/mL) and recombinant human sRANKL (100 ng/mL; both from PeproTech). Media was replaced every 72 hours.

For monocyte derived macrophage (mD-Mφ) differentiation, monocytes were plated at a density of 2 × 10^6^/ml in DMEM, supplemented with penicillin (100 U/ml), streptomycin (100 μg/ml), and gentamicin (50 μg/ml). After 2 h, the media were substituted with media containing 10% heat-inactivated FBS (Life Technologies, Grand Island, NY, USA) and rhM-CSF (50 ng/ml; PeproTech, Rocky Hill, NJ, USA). For monocyte derived DC (mD-DC), monocytes were cultured in RPMI 1640, supplemented with rhGM-CSF (1000 U/ml) and rhIL-4 (500 U/ml; both from PeproTech). Media were replaced every 72 h. At day 7, cells were harvested and differentiation confirmed by flow cytometric analysis of CDw93, CD68, CD209, CD1a, CD11b, and CD11c expression. Additional confirmation of mD-Mφ & mD-DC phenotype can be found in our previous publications^11^,^12^. For macrophage and DC conversion to OC, media was replaced with the same OC-differentiating medium described above with replenishment every 72 hrs.

### OC staining and microscopy

Differentiation was assessed by staining cells for tartrate resistant acid phosphatase activity and number of nuclei (Hoescht, Invitrogen). Lysosomes and mitochondria were stained using LysoTracker and MitoTracker, respectively (Invitrogen). For fluorescent imaging, the EVOS^®^ FL Cell Imaging System (Life Technologies) was used. TRAP staining was performed at 37 C using 10 mg Naphtol AS-MX phosphate, 1 mL dH2O, 0.2M sodium acetate solution, 0.2M acetic acid solution, 0.3M sodium tartrate, 0.1M acetate buffer and TRAP buffer (pH = 5). Modified Giemsa stain (Romanowsky stain variant) was purchased as Diff-Quik (Polysciences, Inc.). Multi-nucleated cells were counted using software supplied as part of the imaging system. In brief, cells were cultured in 96-well plates, the center of each well aligned directly above the objective turret, and a 50 μm × 50 μm grid overlaid using the imaging software. Nuclei and cells present within (in whole or in part) an area of 1600 μm^2^ were tagged and the average number of nuclei per cell was calculated from counts provided by the software. For quantification each sample was run as technical replicates with the average representing the value for the biological replicate. Analysis was performed on the averages from four biological replicates.

### Resorption assays

Collagen degradation was assessed using the Osteolyse^TM^ Assay Kit (Lonza) following the manufacturer’s instructions. Cells were plated on the Osteolyse^TM^ plate and collagen degradation assayed by combining 10 μl of supernatant with 200 μl of Fluorophore Releasing Reagent. Fluorescence was measured over 400 μs with an initial delay of 400 μs (excitation at 340 nm, emission at 615 nm).

### MiRNA profiling

Total RNA was isolated at 0, 3, 6 and 12 days of differentiation using the miRNeasy micro kit (Qiagen) following manufacturer’s instructions. RNA integrity was assessed using the Nanodrop (Thermo Scientific) and 2100 Bioanalyzer (Agilent). MiRNA expression was performed by Exiqon Services using 7th generation microarrays (miRBase v.19). 225 nanograms of total RNA were labeled using the miRCURY LNA^TM^ microRNA Hi-Power Labeling Kit Hy3^TM^/HY5^TM^ and subsequently hybridized onto miRCURY LNA^TM^ microRNA Arrays following the procedures described by the manufacturer. Data normalization was performed by Exiqon using Quantile normalization. Initial analysis was performed using R/bioconductor primarily using the limma package (Exiqon). Expression analysis of variance over time was performed with p values adjusted using the Benjamini Hochberg method and identified genes subjected to the Turkey’s ‘Honest Significant Difference’ test. Array data was in compliance with MIAME guidelines and deposited in the Gene Expression Omnibus (GEO) public database under the accession number GSE60840.

### Bioinformatic Analysis

Bioinformatic analysis was performed on miRNAs identified as significantly (FDR < 0.05), differentially expressed during monocyte-to-OC differentiation. Of these, only the miRNAs whose altered level of expression was maintained (>72 hrs from initial time-point with significance [FDR > 0.05]) were selected for further analysis. We employed miRWalk to predict the candidate 3′-UTR of genes for miRNA binding sites with the 5 established miRNA-target prediction algorithms^22^. MiRNAs that possessed no predicted targets linked to regulation of OC differentiation and function by Gene Ontology (GO) biological terms (http://www.geneontology.org) were not considered. The remaining miRNAs were then ranked according to the sum of predicted targets, with each predicted target being given a value of 1. Identification of the target by multiple algorithms resulted in a value equal to the number of predictive algorithms (i.e. if the same target was identified by 3 of the 5 algorithms then it was given the value of 3). The top-ranked miRNAs were selected for further investigation and functional analysis.

### RT-PCR analysis

Five hundred nanograms of total RNA was used for first strand cDNA synthesis using the miScript RT II kit (Qiagen) according to the manufacturer’s instructions. Expression profiles of miR-223, miR-34a-5p and miR-142-3p were examined by miScript PCR assays and SYBR Green Master Mix (both from Qiagen). Real-time PCR was performed on a StepOne 7500 thermocycler (Applied Biosystems). Expression levels were normalized with respect to RNU6 (Qiagen). PKCα mRNA levels were analyzed using a StepOnePlus Real-Time PCR System (Applied Biosciences). Expression levels were normalized with respect to GAPDH.

### Transient miRNA/siRNA transfection

MirScript miRNA 142-3p mimic and inhibitor were purchased from Qiagen. AllStars negative mimic (Qiagen) was used as control. Gene specific and control siRNA for PKCα knockdown were purchased from Sigma-Aldrich. Transient transfections were performed using Lipofectamine 2000 (Life Technologies) according to manufacturer’s instructions. Cells were transfected with mimics or inhibitors at a final concentration of 100 nM. Red siGLO oligonucleotides (Thermo Fisher Scientific) were used to determine transfection efficiency (S2 Table). Viability was assessed by flow cytometry (S3 Table).

### Western blots

Cells were lysed in cell lysis buffer (Cell Signaling Technology) supplemented with protease inhibitors (Roche). Protein content was estimated using the Bradford assay (BioRad Laboratories). Equal amounts of protein were resolved in 10% Mini-PROTEAN^®^ TGX™ (BioRad) gels and electrotransferred to nitrocellulose membranes (GE Healthcare). Membranes were blocked with 5% skimmed milk for 2 hrs and incubated with primary antibodies against PKCα or GAPDH (1:1000; BD Biosciences) overnight at 4 C. The blots were washed with PBS 0.1% Tween20 thrice before incubating with secondary antibody (1:10,000) for 1 hr at room temperature. Blots were washed with PBS 0.1% Tween20 thrice and protein detection was performed using enhanced chemiluminescence (GE Healthcare). ImageJ software (http://rsbweb.nih.gov/ij/) was used to quantify the results. The values for each lane were normalized with respect to endogenous control GAPDH.

### Viability assays

Cell viability was determined using the CellTiter^®^ 96 AQueous Cell Proliferation Assay Kit (Promega) according to manufacturer’s instructions. In brief, 20 μl of reagent was added to each well of a 96-well plate containing OC and 200 μl of culture medium, incubated for a further 2 hrs, and absorbance at 490 nm was recorded using an ELISA plate reader. In certain experiments viability was also quantified by flow cytometry using the LIVE/DEAD® Fixable Violet Dead Cell staining kit (Invitrogen).

### Statistical Analysis

For miRNA expression, p values were calculated by ANOVA. P values were adjusted using the Benjamini Hochberg method. In brief, p values were sorted and ranked (smallest value = 1, second smallest = 2, largest = N), each p value was multiplied by N and then divided by its assigned rank to give the adjusted p values (FDR). MiRNAs with FDR < 0.05 were considered significant. For ELISA and CellTiter 96^®^ AQueous One Solution data, p values were calculated using the Student’s t-test. Statistical analysis of data was performed using GraphPad Prism software (GraphPad Software, Inc.). Results are presented as mean + standard deviation (SD) or ±SEM from 3 or more biological donors. Data from each individual donor is the mean of 2 technical replicates. A p value of <0.05 is considered statistically significant.

### Ethical Statement

All methods were performed in accordance with approved guidelines. The study and all experimental protocols were approved bythe University of Illinois Institutional Review Board. Informed consent was provided by all subjects prior to participating in the study.

## Additional Information

**How to cite this article**: Fordham, J. B. *et al*. MiR-142-3p is a sRANKL-dependent inducer of cell death in osteoclasts. *Sci. Rep.* **6**, 24980; doi: 10.1038/srep24980 (2016).
